# Supplementary material for: The association between perceived psychosocial support and resilience among Venezuelan migrant women: A secondary analysis of cross-sectional data from 2022
Source: PLOS Ment Health. 2025 Oct 8;2(10):e0000273. doi: 10.1371/journal.pmen.0000273 (PMC12798633; doi:10.1371/journal.pmen.0000273)
Supplement: S1 Appendix — (DOCX) [file pmen.0000273.s001.docx]

**Appendix 1. Survey Questions and Their Coding**

| **Variable** | **Survey Question** | **Available Answers** | **Modifications** |
| --- | --- | --- | --- |
| Perceived Psychosocial Support  (Exposure) | *The woman/girl in the shared story was…* | Continuous slider from “Provided with absolutely no supports / services” to “Provided with too many supports / services” | **Tertiles**  Bottom  Middle  Top |
| Psychological Resilience (Outcome) | *At this time, I am able to cope with the challenges I face:* | - All The Time  - Most Of The Time  - Some Of The Time  - Never  - Prefer Not To Say | **Dichotomized**  “All the time”  “Not all the time” |
| Age | *What is your age?* | Any whole number | Unchanged |
| Ethnicity | *Which of the following groups, if any, did the woman/girl in the story identify with and was most relevant to her experience shared in the story (choose only 1)?* | - Mestiza  - Afro Descendant  - Indigenous  - Prefer Not To Say/Not Sure  - Did Not Identify With Any Of These Groups  - Other | Unchanged |
| Having a Child | *How many children do you have (choose only 1)?* | - 0  - 1 – 2  - 3 Or More  - Prefer Not To Say | **Dichotomized**  ‘No’  ‘Yes’ |
| Having a Partner | *What is your marital status (choose only 1)?* | - Married / In A Union  - Divorced / Separated  - Widowed  - Single, Never Married  - Prefer Not To Say | **Dichotomized**  “Married/In a Union”  “Not Married/Not in a Union” |
| Identifying as LGBTQ+ | *Did the woman or girl in the shared story identify as LGBTQ+ (choose only 1)?* | - Yes  - No  - Prefer Not To Say/Not Sure | Unchanged |
| Length of Displacement | *How long ago did you leave your home in Venezuela (choose only 1)?* | - < 1 Year  - 1 – 3 Years  - 3 – 5 Years  - > 5 Years  - Prefer Not To Say / Not Sure | Unchanged |
| Miscellaneous Health Issues | *Which of the following groups, if any, did the woman/girl in the story identify with and was most relevant to her experience shared in the story (choose only 1)?* | - Having A Disability  - Experiencing Mental Health Problems  - Use of Alcohol And Drugs  - Did Not Identify With Any Of These Groups | Unchanged |
| Relative Wealth | *Relative to the wealth level of others in the community, was the woman/girl in your story (choose only 1)…* | - Very Poor  - Poor  - Average  - Wealthy  - Very Wealthy  - Prefer Not To Say / Not Sure | ‘Wealthy’ and ‘Very Wealthy’ were combined with ‘Average’ into ‘Average or Above’ due to small cell sizes |
