# Supplementary material for: The association between perceived psychosocial support and resilience among Venezuelan migrant women: A secondary analysis of cross-sectional data from 2022
Source: PLOS Ment Health. 2025 Oct 8;2(10):e0000273. doi: 10.1371/journal.pmen.0000273 (PMC12798633; doi:10.1371/journal.pmen.0000273)
Supplement: Attachment — Submitted filename: Response to Reviewers.docx [file pmen.0000273.s006.docx]

Review Comments to the Author

Reviewer #1: I would like to thank you for the opportunity to review this paper. It is a relatively under-studied topic within mental health of forcibly displaced communities, especially women. Thus, it is a pleasure to see such as big sample size and an innovative approach to investigate the contextualized resilience. Please see my comments for consideration below. – Thank you for taking the time to review our manuscript and support our work.

Abstract:

1. I’m not sure if the sentence “resilience protests against these threats..” is correct. The authors refer to migration experiences including sexual exploitation and trafficking as threats and resilience protects against these threats. Forcibly displaced people are resilient despite experiencing these threats/difficulties. I suggest that the authors revise this sentence. – Thank you for pointing out this phrasing. We adjusted the sentence to indicate that resilience protects against the *impacts* of stressors, rather than the occurrence of the stressors themselves.
2. Migrants and displacement are used together. It might be good to clarify whether the study includes Venezuelan migrants or Venezuelan forcibly displaced migrants (voluntary vs. involuntary nature of the migration). – Thank you for helping improve the consistency of our writing. We adjusted the wording throughout the manuscript to uniformly use the broader term ‘migrant’ as the survey could not elucidate the nature of the participants’ migrations (voluntary vs. involuntary, refugee vs. migrant, etc.).
3. What does “fully resilient” mean? – We agree this term was confusing without the additional context provided in the body of the text. We removed ‘fully’ and welcome the readers to review the ‘Introduction’ and ‘Methods’ sections for more detailed explanations of what constitutes resilience within our study.
4. The authors stated that they are interested in the relationship between PSS and resilience, but the results do not include the result of that assumed relationship. They, then, concluded that this study showed PSS as important for resilience. I think they need to include the measures that they used for these two constructions in the study, PSS and resilience in the Abstract. – We included a sentence in the Abstract explaining how PPS and resilience were conceptualized in the survey.

Introduction:

1. I suggest that the authors use forced displacement/forcibly displaced or refugees instead of migrants in the Manuscript. – Please refer to our response to Comment #2 in the Abstract section above.
2. Please include a reference for this sentence, Line 64: “Combined, these factors compound the risk of mental health issues developing..” – After restructuring the first two paragraphs to address the comment below, this sentence no longer appears.
3. The first two paragraphs repeat each other. Especially, the Lines 57-63 with the Lines 67-80. These two paragraphs can be combined and the context can be condensed. – Thank you for helping make our Introduction more concise. We restructured this section and removed overlapping ideas.
4. It is hard to follow the progression from greater risk of mental health due to forced displacement to resilience and state resilience as critical protective factor for mental health. From my perspective, despite all these challenges and increased risk for mental health and health related difficulties, most forcibly displaced communities do not develop mental health problems. In fact, they mostly are resilient. I would definitely pitch the story from the strength perspective, not the deficit. It also depends on how the authors define resilience, is it an outcome or a predictor? –We appreciate this encompassing feedback. Through restructuring our Introduction, we believe this progression is clearer. We also adjusted our wording throughout to better convey a strengths-based perspective with respect to how migrants manage adversity. The studies suggested were interesting to read and did indeed help focus our introduction of resilience. Thank you.

There are resilience-promoting factors such as social support predicting resilience outcomes, which are better mental health/health outcomes than expected.

I strongly recommend the authors to read the following publications to help with their conceptualization: Thank you – these articles were helpful

Ungar, M., & Theron, L. (2020). Resilience and mental health: How multisystemic processes contribute to positive outcomes. The Lancet Psychiatry, 7(5), 441-448.

Schäfer, S. K., Supke, M., Kausmann, C., Schaubruch, L. M., Lieb, K., & Cohrdes, C. (2024). A systematic review of individual, social, and societal resilience factors in response to societal challenges and crises. Communications Psychology, 2(1), 92.

Ciaramella, M., Monacelli, N., & Cocimano, L. C. E. (2022). Promotion of resilience in migrants: a systematic review of study and psychosocial intervention. Journal of immigrant and minority health, 24(5), 1328-1344.

5. Reference needed for Line 95-96 & Line 110-113. – For 95-96, we added a number of references (page 5). For 110-113, we restructured the section and included several references (page 6).
6. The authors mentioned that the Venezuelan refugee crisis is different from the other crisis settings. However, there is no contextual information about in what ways it is different. – Thank you for the suggestion. We added religion and degree of armed conflict as examples (page 7).
7. Overall, I think the Introduction Section can benefit from clarification and refinement. The key issues are: 1) resilience and mental health are used together and interchangeably throughout the manuscript, the outcome variable is not clear. 2) the authors mentioned the importance of understanding the role of contextual factors on shaping resilience and social support, but it wasn’t stated as one of the objectives of the study.
After addressing these issues, I believe the Introduction will be strengthened by clearly defining the key concepts of the investigation and study objectives. – Thank you for the comprehensive feedback. We implemented the following changes in response: A) we altered our wording throughout to distinguish resilience from mental health, B) it should be clearer that resilience is our outcome variable after the changes we made to the structure and word choice, C) we moved our discussion of the importance of context to later in the manuscript so as to not imply that our study delves deeply into this topic, D) the key concepts and objectives of our study should be more clearly communicated in the rewritten Introduction

Methods:

1. The Sense-Making methodology is interesting and seems to address some pitfalls of the qualitative and quantitative methodologies. Has it been used in the forced displacement field before? – Yes it has! We added a few references to inform the reader of this (page 9).
2. The respondents shared stories and answered the PSS question about themselves or other women/girls they might have known. On the other hand, coping ability, the outcome variable, was targeted at the respondent herself. How did the authors address the difference between the differential focus of these questions? What was their analytical approach for the answers for the stories not about the respondent herself? – To address this discrepancy, we limited our analysis to participants whose micro-narratives were autobiographical. To communicate this, we added statements at the ends of the ‘Sampling’ and ‘Survey’ sections (pages 8 and 10).
3. Since the authors operationally defined resilience with the coping item, they need to provide a background in the Introduction that they would consider coping as the proxy measure of resilience. – This suggestion is greatly appreciated. We added half a paragraph briefly outlining coping and how it relates to our study. We felt this information fit best in the ‘Variables’ section, as this is where we discussed the specific survey question utilized (page 11).

4. Please include the name of the categories of PSS following categorizing the response options into tertiles, Line 200-201. – Thank you for suggesting this additional clarification. We added the information in parentheses (page 10).
5. There needs to be a justification for categorizing the coping question into two. How did the authors consider “some of the time” or “never” as those with potential improvement? – On further reflection, we agree that this wording was a little confusing. We adjusted the phrasing to instead emphasize *benefiting* from improvement, rather than possessing the physical capacity to improve (page 11).
6. Please include the name of the eight potential covariates, Line 211. Were they all categorical variables so that the authors used chi-square to examine the association between each of these variables and resilience (coping)? – Age was a continuous variable while the rest were categorical. We had changed how the age variable was handled following a discussion with a biostatistician, and we evidently had not caught all the changes we needed to make in our write-up, so thank you for flagging this. It has been corrected. Regarding the names of the confounders, we added this information in parentheses (page 11).
7. Provide a reference for the choice of 0.20 as a threshold, Line 221. – No reference was included since, unfortunately, in our review of the literature we were unable to find sufficiently relevant studies that utilized regression modeling and a refinement strategy that relied on p-value cut-offs (we settled on 0.20 following discussions with a biostatistician). We added an explanation to the section to provide the reader with the needed context (page 12).

Results:

1. The authors discussed the importance of contextualizing the experiences of Venezuelan women/girls; however, they haven’t used the relevant variables in the parent questionnaire, such as the star question, in their analyses. Which aspect of their analyses provided the contextualized experiences of the participants? – Please see our response to comment #7 in the ‘Introduction’ section above.

Discussion:

1. The reference 62 that the authors used to compare their results is with the Syrian refugee children, which does not provide a proper comparison. Two-thirds of the sample indicated probable resilience, and this estimate is comparable to those found in the extant literature with forcibly displaced populations. Most of the studies showed that approximately one third of the forcibly displaced individuals develop mental health issues (For instance: Blackmore, R., Boyle, J. A., Fazel, M., Ranasinha, S., Gray, K. M., Fitzgerald, G., ... & Gibson-Helm, M. (2020). The prevalence of mental illness in refugees and asylum seekers: A systematic review and meta-analysis. PLoS medicine, 17(9), e1003337.) – Thank you for this comment, and we found Blackmore 2020 to be an insightful read. We adjusted this part of the section to discuss in greater detail the difficulties associated with directly comparing our results with previous findings. Blackmore 2020 and Usta 2025 were included as supporting examples (page 19).
2. Line 374-377: The program components are not clear. Why workshops and why four weeks as the program duration? Please mention some of the evidence-based programs or strategies used with this population or a similar population to promote resilience and coping. – To address this, we adjusted our phrasing at several points, included a second systematic review, and discussed in more detail how previous interventions have been designed (page 22).
3. Line 395-396: Please provide references for “few studies”. – Thank you for this comment. However, as this point is not derived from any individual study but, rather, is representative of our own experiences in examining the literature, we felt it was best to not include any reference. However, we adjusted this sentence to better communicate the situation (page 23).
4. Overall, the authors provided the summary of their findings in the first two pages of their Discussion, however, the comparison of the resilience predicting factors with the extant literature is lacking in this section. How do the current findings complement/contradict the extant literature? – We agree these pages would benefit from additional discussion. Unfortunately, several of the variables are severely understudied in the literature, and thus minimal applicable information is available on them. In general, we added more results from previous studies and, where applicable, an explanation of any gaps in the literature.
