# Supplementary material for: The association between perceived psychosocial support and resilience among Venezuelan migrant women: A secondary analysis of cross-sectional data from 2022
Source: PLOS Ment Health. 2025 Oct 8;2(10):e0000273. doi: 10.1371/journal.pmen.0000273 (PMC12798633; doi:10.1371/journal.pmen.0000273)
Supplement: Attachment — Submitted filename: Response_to_Reviewers_auresp_2.docx [file pmen.0000273.s007.docx]

**Reviewer #2:**

Thank you for the opportunity to review this new version of the manuscript. Here's my detailed feedback and recommendations for improvement. This manuscript presents research on the resilience of migrant women from Venezuela in some South American countries. While the topic is certainly relevant and the study aims to fill important gaps, there are still key areas that require significant clarification and expansion before it can be considered for publication. – Thank you for taking the time to review our manuscript and help strengthen our discussion.

The authors need to explicitly clarify the rationale for selecting participants aged "14+". This is crucial, as the legal implications of including minors (14-17 years old) must be thoroughly addressed, particularly concerning informed consent and their emancipation status within the specific legal frameworks of Brazil, Ecuador, and Peru. If legal emancipation was a criterion, it should be clearly stated and defined according to each country's laws or IOM definition. – Thank you for helping to improve our clarity. We included more details on the rationale in the ethical considerations section of the methods on page 13.

One of the main variables is displacement duration, yet the participants are broadly described as migrants. The manuscript must differentiate between various legal and social categories of participants, such as refugees, asylum seekers, economic migrants, internally displaced persons, irregular migrants, or regular migrants. This distinction is vital because these statuses are directly linked to varying levels of vulnerability, access to resources, and consequently, mental health outcomes and resilience mechanisms. The current ambiguity significantly hinders the interpretation of findings related to "displacement duration." This was mentioned in the first review but was just reframed as migrant and this does not solve the problem. – We agree that this was a significant limitation with the available data. In the ‘Strengths and Limitations’ section on page 26, we added an explanation of why data on this variable was not captured in the source study, in addition to a more detailed discussion of how this impacted interpretation of the results.

The study involved interviews in different cities across Brazil, Ecuador, and Peru, but it largely omits a detailed discussion of the distinct socio-economic, political, and cultural realities within and between these countries. These contextual differences could significantly influence participants' experiences and resilience. While Lima is briefly mentioned, a more comprehensive analysis of these contextual factors is essential to enrich the findings and provide a nuanced understanding of the phenomenon under study. Such a comparative analysis would considerably strengthen the document. – Thank you for the suggestion. The ‘Discussion’ section discussing ethnicity on page 21 now includes a brief illustration of how each nation has supported, or struggled to support, Venezuelan migrants.

The authors' reliance on their own previous publications for methodological discussions is a significant limitation. It is imperative to incorporate a broader range of external academic literature on the research methodologies, data analysis techniques (especially given the mention of different analytical approaches), and theoretical frameworks relevant to resilience studies or at least migration studies. Citing external, foundational texts would enhance the methodological rigor and scholarly credibility of the manuscript. Furthermore, while software company documentation might be useful for technical details, it should not serve as a primary methodological source and should be minimized or recontextualized within broader methodological discussions. – We agree that the scholarly credibility of this section required improvement. Thus, we have included several other sources that discuss/utilize the Sensemaking methodology (Souto, et. al 2012, Savolainen 1993, and Mamykina 2015). However, we felt it was still important to retain the three references to Dr. Bartel’s previous works as they constitute the most direct parallels to our current analysis.

The statement on "68% of resilience in migrants" found on P22 L395, along with its associated citation, requires immediate clarification. The authors must explain the source, methodology, and context of this statistic. Is it a prevalence rate from a specific study, a general finding, or a measure derived from their own data? The current presentation is vague and potentially misleading. – We assume the citation referenced here is Blackmore 2020 as line 395 did not correspond to the quoted statement in our version of the document. We therefore adjusted the wording of this example to better contextualize the results of the meta-analysis.

Despite focusing on women participants, the manuscript does not consistently apply a clear gender perspective throughout the analysis and discussion. The authors should elaborate on how gender shapes the experiences of migration/displacement, resilience, and mental health for these women. Furthermore, the discussion regarding the exclusion of participants who do not identify as women, while simultaneously mentioning "LGBT+" as a variable, is confusing. The authors must clarify whether transwomen were included in the sample and, if so, how their experiences were considered within the "women" framework. They also need to explain the exact number and experiences of any LBT (Lesbian, Bisexual, Transgender) participants within the "women" sample, if applicable, and why "LGBT+" was initially considered as a variable if the study exclusively focused on women. The theoretical and analytical implications of these distinctions need to be thoroughly addressed. – We greatly appreciate the comprehensive feedback. We expanded on our discussion of the experiences of female Venezuelan migrants in the ‘Introduction’ and ‘Discussion’ sections. Additionally, as the source study did not gather details beyond whether participants self-identified as LGBTQ+ and as women, we lack the data to distinguish between cisgender women and transgender women. However, we did clarify in the ‘Sampling’ subsection on page 9 that both cisgender and transgender women were eligible to participate in the original study.

The manuscript includes "disability" as a variable but does not define what this means within the context of the study or for the participating women. A deeper exploration of how disability intersects with migration/displacement and influences resilience for these women is warranted. This could include, for example, access to services, social support, and specific challenges faced. – Thank you for identifying this inadequacy. Since research at the intersection of resilience, migrant health, and disabilities is sparse, on page 22-23, we added a brief discussion on a broad set of several potentially resilience-modulating factors that would be likely be relevant to the experiences of Venezuelan migrant women. Additionally, to improve clarity regarding how disability was defined, we added “(as defined by the individual)” when the disability variable is first mentioned near the beginning of the ‘Descriptive statistics’ subsection on page 14.

The concluding statement asserting that "This study is also distinct in being the only of its kind to investigate the resilience of migrants navigating a contemporary humanitarian crisis in South America. Furthermore, based on our review of the literature, resilience studies that focus exclusively on migrant women are uncommon, and those that do often limit their samples to female subgroups, such as Sim’s work with Syrian mothers" is a significant overstatement. The authors must conduct a more thorough and comprehensive literature review, particularly considering studies published in Spanish and Portuguese, which are abundant in this field and region. The authors should revise this statement to accurately reflect the existing body of literature and articulate the specific unique contribution of their study within that broader context. – Thank you for your feedback. On page 25, we added two studies focused on the resilience of Venezuelan migrant women and adjusted our phrasing (Seff 2025 and Bautista 2025).

By addressing these points, the authors can significantly enhance the rigor, clarity, and scholarly contribution of their manuscript.

Thank you again for taking the time to provide this valuable feedback.
